# Supplementary material for: Heparin-based hydrogel scaffolding alters the transcriptomic profile and increases the chemoresistance of MDA-MB-231 triple-negative breast cancer cells
Source: Biomater Sci. 2020 Feb 13;8(10):2786–96. doi: 10.1039/c9bm01481k (PMC7497406; doi:10.1039/c9bm01481k)
Supplement: Supplementary file 2 [file BM-008-C9BM01481K-s002.zip › Supplementary File 4/HP-B hydrogel + EGF (s)vControl/Pathways/my_analysis.Gsea.1545201170987/HALLMARK_COMPLEMENT.html]

Details for gene set HALLMARK\_COMPLEMENT[GSEA]

|  || Dataset | expr.class.cls#HYDEGF\_versus\_CONTROL.class.cls#HYDEGF\_versus\_CONTROL\_repos |
| Phenotype | class.cls#HYDEGF\_versus\_CONTROL\_repos |
| Upregulated in class | CONTROL |
| GeneSet | HALLMARK\_COMPLEMENT |
| Enrichment Score (ES) | -0.486987 |
| Normalized Enrichment Score (NES) | -2.2205427 |
| Nominal p-value | 0.0 |
| FDR q-value | 0.0 |
| FWER p-Value | 0.0 |
Table: GSEA Results Summary

  

Fig 1: Enrichment plot: HALLMARK\_COMPLEMENT      
 Profile of the Running ES Score & Positions of GeneSet Members on the Rank Ordered List

  

| PROBE | DESCRIPTION (from dataset) | GENE SYMBOL | GENE\_TITLE | RANK IN GENE LIST | RANK METRIC SCORE | RUNNING ES | CORE ENRICHMENT || 1 | RASGRP1 | na |  |  | 87 | 2.987 | 0.0179 | No |
| 2 | EHD1 | na |  |  | 1297 | 1.516 | -0.0342 | No |
| 3 | KCNIP2 | na |  |  | 1546 | 1.411 | -0.0366 | No |
| 4 | RAF1 | na |  |  | 1584 | 1.399 | -0.0280 | No |
| 5 | COL4A2 | na |  |  | 1989 | 1.269 | -0.0397 | No |
| 6 | KCNIP3 | na |  |  | 2255 | 1.193 | -0.0446 | No |
| 7 | GATA3 | na |  |  | 2562 | 1.112 | -0.0523 | No |
| 8 | ERAP2 | na |  |  | 2691 | 1.082 | -0.0509 | No |
| 9 | AKAP10 | na |  |  | 2879 | 1.032 | -0.0530 | No |
| 10 | IRF7 | na |  |  | 2931 | 1.020 | -0.0480 | No |
| 11 | F3 | na |  |  | 2957 | 1.016 | -0.0417 | No |
| 12 | ZFPM2 | na |  |  | 3638 | 0.878 | -0.0707 | No |
| 13 | BRPF3 | na |  |  | 3839 | 0.839 | -0.0749 | No |
| 14 | DGKH | na |  |  | 3986 | 0.814 | -0.0765 | No |
| 15 | SH2B3 | na |  |  | 4387 | 0.752 | -0.0918 | No |
| 16 | DOCK9 | na |  |  | 4674 | 0.710 | -0.1015 | No |
| 17 | MAFF | na |  |  | 4719 | 0.703 | -0.0985 | No |
| 18 | PCLO | na |  |  | 4841 | 0.680 | -0.0998 | No |
| 19 | JAK2 | na |  |  | 4877 | 0.671 | -0.0965 | No |
| 20 | RCE1 | na |  |  | 5158 | 0.628 | -0.1065 | No |
| 21 | TMPRSS6 | na |  |  | 5176 | 0.624 | -0.1027 | No |
| 22 | C4BPB | na |  |  | 5314 | 0.603 | -0.1054 | No |
| 23 | SIRT6 | na |  |  | 5366 | 0.594 | -0.1036 | No |
| 24 | MMP15 | na |  |  | 5812 | 0.531 | -0.1229 | No |
| 25 | PRDM4 | na |  |  | 5895 | 0.518 | -0.1234 | No |
| 26 | PRSS36 | na |  |  | 5985 | 0.504 | -0.1242 | No |
| 27 | GP1BA | na |  |  | 6003 | 0.502 | -0.1214 | No |
| 28 | PLG | na |  |  | 6144 | 0.483 | -0.1251 | No |
| 29 | PCSK9 | na |  |  | 6303 | 0.463 | -0.1299 | No |
| 30 | STX4 | na |  |  | 6377 | 0.454 | -0.1303 | No |
| 31 | RNF4 | na |  |  | 6805 | 0.396 | -0.1497 | No |
| 32 | CTSH | na |  |  | 6847 | 0.390 | -0.1490 | No |
| 33 | ME1 | na |  |  | 7136 | 0.354 | -0.1614 | No |
| 34 | CDK5R1 | na |  |  | 7158 | 0.350 | -0.1599 | No |
| 35 | GNB2 | na |  |  | 7318 | 0.327 | -0.1658 | No |
| 36 | RHOG | na |  |  | 9155 | 0.108 | -0.2613 | No |
| 37 | FDX1 | na |  |  | 9724 | 0.046 | -0.2907 | No |
| 38 | SERPINB2 | na |  |  | 9903 | 0.024 | -0.2999 | No |
| 39 | PRKCD | na |  |  | 10287 | -0.019 | -0.3199 | No |
| 40 | CEBPB | na |  |  | 10406 | -0.036 | -0.3258 | No |
| 41 | GCA | na |  |  | 10593 | -0.049 | -0.3352 | No |
| 42 | CALM3 | na |  |  | 10601 | -0.050 | -0.3352 | No |
| 43 | SRC | na |  |  | 10830 | -0.082 | -0.3465 | No |
| 44 | IRF1 | na |  |  | 11060 | -0.115 | -0.3577 | No |
| 45 | PIM1 | na |  |  | 11181 | -0.119 | -0.3631 | No |
| 46 | DYRK2 | na |  |  | 11183 | -0.119 | -0.3622 | No |
| 47 | DOCK10 | na |  |  | 11304 | -0.137 | -0.3675 | No |
| 48 | F8 | na |  |  | 11442 | -0.152 | -0.3735 | No |
| 49 | HSPA1A | na |  |  | 11480 | -0.160 | -0.3743 | No |
| 50 | PHEX | na |  |  | 11721 | -0.190 | -0.3854 | No |
| 51 | PRSS3 | na |  |  | 11733 | -0.192 | -0.3846 | No |
| 52 | ANG | na |  |  | 12069 | -0.228 | -0.4004 | No |
| 53 | GPD2 | na |  |  | 12156 | -0.238 | -0.4031 | No |
| 54 | CFH | na |  |  | 12170 | -0.240 | -0.4020 | No |
| 55 | GRB2 | na |  |  | 12300 | -0.261 | -0.4068 | No |
| 56 | CASP1 | na |  |  | 12497 | -0.286 | -0.4150 | No |
| 57 | FYN | na |  |  | 12769 | -0.326 | -0.4267 | No |
| 58 | PIK3CG | na |  |  | 12934 | -0.335 | -0.4328 | No |
| 59 | TIMP2 | na |  |  | 13099 | -0.353 | -0.4388 | No |
| 60 | ADAM9 | na |  |  | 13107 | -0.354 | -0.4365 | No |
| 61 | USP16 | na |  |  | 13158 | -0.362 | -0.4364 | No |
| 62 | LRP1 | na |  |  | 13236 | -0.376 | -0.4376 | No |
| 63 | KIF2A | na |  |  | 13613 | -0.427 | -0.4541 | No |
| 64 | PFN1 | na |  |  | 13723 | -0.445 | -0.4565 | No |
| 65 | CP | na |  |  | 13757 | -0.449 | -0.4549 | No |
| 66 | CBLB | na |  |  | 13811 | -0.460 | -0.4542 | No |
| 67 | LGMN | na |  |  | 13973 | -0.484 | -0.4590 | No |
| 68 | PLAUR | na |  |  | 14166 | -0.503 | -0.4653 | No |
| 69 | USP15 | na |  |  | 14247 | -0.513 | -0.4656 | No |
| 70 | NOTCH4 | na |  |  | 14269 | -0.516 | -0.4629 | No |
| 71 | PLSCR1 | na |  |  | 14338 | -0.526 | -0.4625 | No |
| 72 | PPP2CB | na |  |  | 14561 | -0.566 | -0.4699 | No |
| 73 | GNB4 | na |  |  | 14652 | -0.581 | -0.4702 | No |
| 74 | DOCK4 | na |  |  | 14796 | -0.602 | -0.4732 | No |
| 75 | XPNPEP1 | na |  |  | 14895 | -0.618 | -0.4737 | No |
| 76 | USP8 | na |  |  | 15004 | -0.633 | -0.4746 | No |
| 77 | DPP4 | na |  |  | 15076 | -0.647 | -0.4735 | No |
| 78 | GMFB | na |  |  | 15204 | -0.670 | -0.4751 | No |
| 79 | CDA | na |  |  | 15208 | -0.671 | -0.4702 | No |
| 80 | APOBEC3G | na |  |  | 15436 | -0.707 | -0.4768 | No |
| 81 | CTSD | na |  |  | 15631 | -0.749 | -0.4814 | Yes |
| 82 | PIK3CA | na |  |  | 15657 | -0.755 | -0.4770 | Yes |
| 83 | CASP7 | na |  |  | 15674 | -0.757 | -0.4721 | Yes |
| 84 | RABIF | na |  |  | 15681 | -0.758 | -0.4668 | Yes |
| 85 | GNAI3 | na |  |  | 15764 | -0.777 | -0.4652 | Yes |
| 86 | PDGFB | na |  |  | 15834 | -0.789 | -0.4629 | Yes |
| 87 | CTSC | na |  |  | 15844 | -0.790 | -0.4575 | Yes |
| 88 | USP14 | na |  |  | 16065 | -0.837 | -0.4627 | Yes |
| 89 | PRCP | na |  |  | 16204 | -0.871 | -0.4634 | Yes |
| 90 | CASP9 | na |  |  | 16279 | -0.893 | -0.4606 | Yes |
| 91 | CSRP1 | na |  |  | 16302 | -0.898 | -0.4550 | Yes |
| 92 | SERPINA1 | na |  |  | 16347 | -0.909 | -0.4505 | Yes |
| 93 | LYN | na |  |  | 16413 | -0.926 | -0.4469 | Yes |
| 94 | ZEB1 | na |  |  | 16483 | -0.942 | -0.4434 | Yes |
| 95 | CXCL1 | na |  |  | 16603 | -0.969 | -0.4424 | Yes |
| 96 | TIMP1 | na |  |  | 16662 | -0.990 | -0.4380 | Yes |
| 97 | PREP | na |  |  | 16698 | -0.999 | -0.4323 | Yes |
| 98 | PLA2G4A | na |  |  | 16752 | -1.011 | -0.4275 | Yes |
| 99 | CFB | na |  |  | 16882 | -1.049 | -0.4264 | Yes |
| 100 | PLAT | na |  |  | 16888 | -1.050 | -0.4188 | Yes |
| 101 | TNFAIP3 | na |  |  | 16896 | -1.053 | -0.4112 | Yes |
| 102 | PPP4C | na |  |  | 17134 | -1.122 | -0.4152 | Yes |
| 103 | DUSP5 | na |  |  | 17173 | -1.134 | -0.4087 | Yes |
| 104 | CPM | na |  |  | 17189 | -1.139 | -0.4009 | Yes |
| 105 | C3 | na |  |  | 17200 | -1.144 | -0.3929 | Yes |
| 106 | GNAI2 | na |  |  | 17280 | -1.174 | -0.3882 | Yes |
| 107 | GNG2 | na |  |  | 17355 | -1.201 | -0.3831 | Yes |
| 108 | C2 | na |  |  | 17358 | -1.202 | -0.3741 | Yes |
| 109 | CASP3 | na |  |  | 17375 | -1.207 | -0.3659 | Yes |
| 110 | LAP3 | na |  |  | 17397 | -1.213 | -0.3579 | Yes |
| 111 | C1R | na |  |  | 17523 | -1.268 | -0.3549 | Yes |
| 112 | PSEN1 | na |  |  | 17614 | -1.300 | -0.3499 | Yes |
| 113 | VCPIP1 | na |  |  | 17692 | -1.328 | -0.3439 | Yes |
| 114 | CASP4 | na |  |  | 17716 | -1.342 | -0.3351 | Yes |
| 115 | APOBEC3F | na |  |  | 17719 | -1.343 | -0.3251 | Yes |
| 116 | LIPA | na |  |  | 17753 | -1.361 | -0.3166 | Yes |
| 117 | HSPA5 | na |  |  | 17766 | -1.367 | -0.3069 | Yes |
| 118 | CALM1 | na |  |  | 17930 | -1.456 | -0.3045 | Yes |
| 119 | PDP1 | na |  |  | 17969 | -1.472 | -0.2955 | Yes |
| 120 | IRF2 | na |  |  | 17974 | -1.475 | -0.2846 | Yes |
| 121 | APOC1 | na |  |  | 18177 | -1.597 | -0.2832 | Yes |
| 122 | IL6 | na |  |  | 18227 | -1.619 | -0.2736 | Yes |
| 123 | ATOX1 | na |  |  | 18310 | -1.694 | -0.2652 | Yes |
| 124 | ANXA5 | na |  |  | 18348 | -1.710 | -0.2543 | Yes |
| 125 | S100A13 | na |  |  | 18364 | -1.725 | -0.2421 | Yes |
| 126 | CTSS | na |  |  | 18454 | -1.799 | -0.2332 | Yes |
| 127 | LTA4H | na |  |  | 18508 | -1.843 | -0.2222 | Yes |
| 128 | LGALS3 | na |  |  | 18514 | -1.848 | -0.2086 | Yes |
| 129 | CTSB | na |  |  | 18524 | -1.856 | -0.1951 | Yes |
| 130 | CLU | na |  |  | 18586 | -1.909 | -0.1839 | Yes |
| 131 | CASP10 | na |  |  | 18591 | -1.910 | -0.1698 | Yes |
| 132 | LAMP2 | na |  |  | 18797 | -2.190 | -0.1641 | Yes |
| 133 | CD46 | na |  |  | 18818 | -2.227 | -0.1484 | Yes |
| 134 | CD59 | na |  |  | 18886 | -2.333 | -0.1344 | Yes |
| 135 | PSMB9 | na |  |  | 18892 | -2.341 | -0.1171 | Yes |
| 136 | CTSO | na |  |  | 18944 | -2.443 | -0.1014 | Yes |
| 137 | DUSP6 | na |  |  | 18966 | -2.496 | -0.0837 | Yes |
| 138 | TFPI2 | na |  |  | 18994 | -2.568 | -0.0658 | Yes |
| 139 | CD55 | na |  |  | 19064 | -2.791 | -0.0485 | Yes |
| 140 | C1S | na |  |  | 19156 | -3.351 | -0.0281 | Yes |
| 141 | KYNU | na |  |  | 19189 | -4.024 | 0.0005 | Yes |
Table: GSEA details [plain text format]

  

Fig 2: HALLMARK\_COMPLEMENT      
 Blue-Pink O' Gram in the Space of the Analyzed GeneSet

  

Fig 3: HALLMARK\_COMPLEMENT: Random ES distribution      
 Gene set null distribution of ES for **HALLMARK\_COMPLEMENT**
